# Functional Nanohybrids and Nanocomposites Development for the Removal of Environmental Pollutants and Bioremediation

**DOI:** 10.3390/molecules27154856

**Published:** 2022-07-29

**Authors:** Giulia Rando, Silvia Sfameni, Maurilio Galletta, Dario Drommi, Simone Cappello, Maria Rosaria Plutino

**Affiliations:** 1 Department of Chemical, Biological, Pharmaceutical and Analytical Sciences (ChiBioFarAm), University of Messina, 98166 Messina, Italy; girando@unime.it (G.R.); mgalletta@unime.it (M.G.); ddrommi@unime.it (D.D.); 2 Institute for the Study of Nanostructured Materials, ISMN—CNR, Palermo, c/o Department of ChiBioFarAm, University of Messina, 98166 Messina, Italy; ssfameni@unime.it; 3 Department of Engineering, University of Messina, 98166 Messina, Italy; 4 Institute for Biological Resources and Marine Biotechnologies, IRBIM-CNR, Spianata San Raineri 86, 98122 Messina, Italy

**Keywords:** nanotechnology, nanomaterials, nanocomposites, nanohybrids, contaminants, environmental remediation, bioremediation

## Abstract

World population growth, with the consequent consumption of primary resources and production of waste, is progressively and seriously increasing the impact of anthropic activities on the environment and ecosystems. Environmental pollution deriving from anthropogenic activities is nowadays a serious problem that afflicts our planet and that cannot be neglected. In this regard, one of the most challenging tasks of the 21st century is to develop new eco-friendly, sustainable and economically-sound technologies to remediate the environment from pollutants. Nanotechnologies and new performing nanomaterials, thanks to their unique features, such as high surface area (surface/volume ratio), catalytic capacity, reactivity and easy functionalization to chemically modulate their properties, represent potential for the development of sustainable, advanced and innovative products/techniques for environmental (bio)remediation. This review discusses the most recent innovations of environmental recovery strategies of polluted areas based on different nanocomposites and nanohybrids with some examples of their use in combination with bioremediation techniques. In particular, attention is focused on eco-friendly and regenerable nano-solutions and their safe-by-design properties to support the latest research and innovation on sustainable strategies in the field of environmental (bio)remediation.

## 1. Introduction

**Figure 1:**
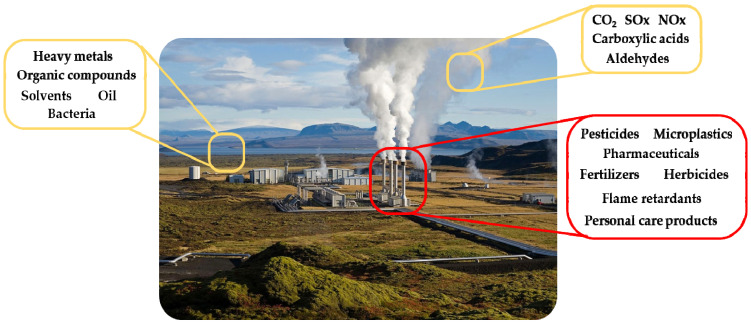
Common (yellow) and emerging (red) worldwide environmental contaminants.

Environmental contaminants and pollutants, deriving from human activity linked to industries, agriculture, waste treatment plants and urban wastewater, are increasing and dangerous, causing adverse effects on human health and earth ecosystems. In this regard, today’s society needs to tackle the remediation of sites contaminated by the most common pollutants, and implement and design new remediation systems due to the presence of new and emerging pollutants that since the last century have been continuously released into the environment by several, new anthropogenic activities (Figure 1) [1,2,3].

Some preventive measurements employed for the protection of ecosystems don’t completely safeguard the environment. In addition, it is necessary to act promptly when environmental emergencies occur (e.g., oil spills), and to eliminate as much as possible the pollutants already present in the environment. 

However, most of the employed current techniques of remediation and waste management require high energy consumption and chemicals that, for example, in the case of wastewater treatment plants or polymeric absorbers/filters for hydrocarbons and pollutants, themselves represent a source of secondary pollution [4].

Nowadays nanotechnologies and new nanomaterials have applications in many sectors due to their properties such as high surface area (surface/volume ratio), size effects, catalytic capacity, and reactivity. In particular, custom-developed nanomaterials may be chemically modified, functionalized and embedded in specific polymers and matrices to produce hybrid materials or nanocomposites with specific chemical, physical and mechanical properties, which may be used for the development of sustainable, advanced and innovative products/techniques for environmental remediation [5,6,7,8,9]. In this regard, useful starting nanomaterials that can be used as functional nanofillers may be classified into: (i) metal nanomaterials (i.e., nanoparticles, NPs, of Pt, Pd, Ni, Ru, Al, Ag, Au, Cu) with characteristic chemical, optical, and electrical properties; (ii) metal oxide nanomaterials (i.e., TiO_2_, Fe_2_O_3_, Al_2_O_3_, ZnO, and SiO_2_), either obtained from sol-gel synthesis or hydrothermal reactions; (iii) bimetallic nanomaterials; (iv) carbon-based nanomaterials (i.e., fullerene, carbon nanotubes CNT, graphene sheets); (v) zeolite and silica-based nanomaterials with a mesoporous structure; (vi) ceramic nanomaterials; (vii) polymeric nanomaterials; (viii) bio-nanomaterials; (ix) metal–organic frameworks, and (x) core–shell nanomaterials [10]. These hybrid organic-inorganic nanomaterials, [11] obtainable by various synthetic routes, and coming from a mixture of two amorphous or crystalline phases [12], can be classified into:

- Composites: i.e., mixtures of materials consisting of a matrix with dispersion at the micrometric level;
- Nanocomposites: i.e., sub-micrometric mixtures (1–100 nm) of materials of a similar nature;
- Hybrids: i.e., sub-micrometric mixtures of materials of a different nature compared to the compound hybrid material;
- Nanohybrid: i.e., atomic or molecular mixtures of different materials with chemical bonds between them.

The transition from hybrid to nano-composite is gradual. A hybrid material in which the constituent units (organic or inorganic) assume the nanometric dimension, can also be considered a nano-composite [13].

In addition, (nano)hybrid materials themselves may be classified according to the interactions between the two phases [14]:

- Class I hybrids: characterized by weak interactions between the phases (van der Waals forces, hydrogen bonds or weak electrostatic interactions);
- Class II hybrids: showing strong interactions (of the first order) between the phases (covalent, ionic bonds).

**Figure 2:**
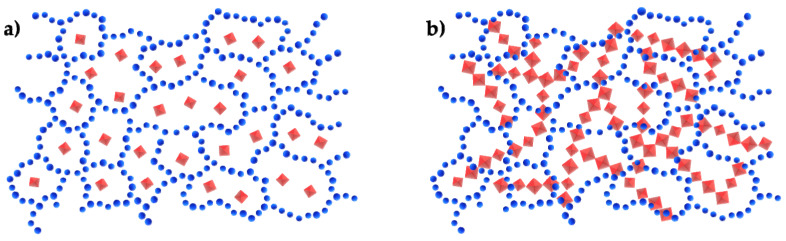
Class I hybrid materials.

If the chemical interactions between the organic and inorganic phases are not strong (Class I hybrids), it is possible to have, as described in Figure 2, a continuous phase that “traps” one dispersed phases (a), or two continuous interpenetrated phases (b).

**Figure 3:**
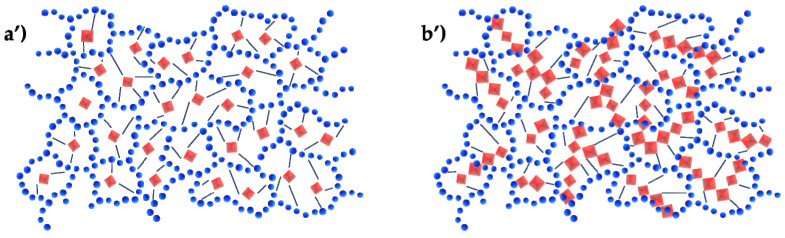
Class II hybrid materials.

If the chemical interactions between the organic and inorganic phases are strong (Class II hybrids), it is possible to have, as described in Figure 3, discrete inorganic units; for example, clusters, covalently linked to a continuous organic phase or vice versa (a’), or two continuous phases linked covalently (b’) [15].

In particular, these classes of nanocomposites and nanohybrids, based on some of the above-mentioned functional nanomaterials (nano-fillers) in combination with organic/inorganic polymeric matrices (obtained by sol-gel techniques [16,17,18,19,20,21], or derived by synthetic or natural organic polymers), due to their mechanical, physical and adsorption properties, can be efficiently employed for the treatment of wastewater, soils and air contaminated by organic substances, heavy metals, hydrocarbons, inorganic salts and radionuclides, and also contaminated by pathogens such as bacteria and viruses [22].

Table 1 lists some pollutants requiring treatment and remediation to safeguard the environment. 

Due to the development of tunable properties and the versatility of different nanomaterials and embedding polymers, nowadays new functional nanohybrids and nanocomposites for pollution remediation are being designed and developed.

Another important aspect of these materials concerns not only their catalytic or photocatalytic ability to degrade contaminants, as in the case of TiO_2_ based nanocomposites [42], but also the possibility of exploiting them for bioremediation. As a matter of fact, they can also act as ideal substrates for bacterial growth as well as stimulants for microorganisms to promote and accelerate the removal of contaminants from the environment [43].

In this regard, bioremediation may be considered a green technology for the restoration of contaminated areas, soils and water (surface and groundwater), by the use of biological agents such as bacteria, fungi, and other organisms or their enzymes. This approach is natural and sustainable, has low cost, and promotes the decomposition and/or removal of inorganic and organic pollutants from contaminated sites. There are two types of bioremediation process, depending on their action: in-situ bioremediation, involving treatment of contaminated substances at the same place, and ex-situ bioremediation, where the contaminated material is treated somewhere else [44,45]. The most common bioremediation technologies involve bioventing, biosparging, land farming, composting, bioaugmentation, and biostimulation [46,47]. There are many examples in the scientific literature regarding the combination of nanotechnologies with bioremediation techniques [48,49], as reported in this review. 

Since it is possible to combine the properties of nanomaterials with ecofriendly approaches for environmental remediation [50], new advances in research in this field are represented by the development of new innovative nanocomposites and hybrids featuring different functional properties, which can be also easily regenerated and reused so that they, themselves, do not become a source of waste or secondary pollution.

This review describes the most recent innovations in environmental recovery strategies for polluted areas based on different nanomaterials, nanocomposites and nanohybrids, with some examples of their use in combination with bioremediation techniques. In particular, it considers nanocomposites and nanohybrids based on:

- metal NPs;
- metal oxide NPs;
- carbon-based nanomaterials;
- silica-based nanomaterials;

We focus attention on eco-friendly and regenerable nanosolutions and their safe-by-design properties (even after their use), to address research and innovation activities towards sustainable strategies in the field of environmental remediation and bioremediation.

## 2. Metal Nanoparticles

### 2.1. Properties of Metal NPs

Metal nanoparticles (MNPs; related to metals or noble metals such as M = Pt, Pd, Ni, Ru, Al, Ag, Au, Cu) are nanomaterials with physical and chemical properties that differ from bulk materials due to their small size and high surface-to-volume ratio. One of the most important characteristics of MNPs is their antibacterial properties and bacterial killing capacity, that occur through different mechanisms such as production of reactive oxygen species, ATP depletion, damage to biomolecules, cation release, and membrane interaction [51]. MNPs have also important optical and electrical properties (extinction, absorption, Rayleigh scattering, Raman scattering, Plasmon Resonances) [52,53] and catalytic properties with a reactivity influenced by the particle size, geometry, composition, oxidation state, and chemical/physical environment [54]. These characteristics allow these nanomaterials to find applications in various sectors such as nanocatalysis, sensing and biosensing, smart textiles [55], biomedicine (molecular diagnostics, imaging, drug delivery and therapeutics) [56,57,58,59], and also in the environmental remediation sector [60]. Different techniques to synthesize MNPs have been developed, such as chemical (e.g., chemical reduction, chemical vapor deposition, photochemical reduction, co-precipitation, thermal decomposition, hydrolysis,) and physical methods (e.g., mechanical milling, laser ablation, vapor deposition, ion sputtering, grinding, flame pyrolysis). These methods can be categorized as bottom-up or top-down methods [61]. 

Green processes for the synthesis of MNPs have also been reported [62], together with their use in the sector of bioremediation and production of biosurfactants. Bioremediation processes, based on the use of biosurfactants that facilitate the solubility of hydrophobic contaminants and remediate contaminated marine sites with a green approach, can be improved by the use of nanoparticles. FeNPs, for example, are of significant importance for stimulation of the production (increased to 80%) of biosurfactants by bacteria such as the marine *Actinobacterium nocardiopsis MSA13A* [63]. 

### 2.2. MNP Composites and Hybrids Applications

MNPs featuring very interesting and useful properties may be used in combination with polymeric matrices for the formation of functional nanocomposites or nanohybrids. to produce the physical properties of the final materials.

In this regard, they can allow the formation of different nanocomposites such as polymer-matrix composites, which consist of isolated nanoparticles finely dispersed in a polymer, composite nanoparticles, such as core/shell nanoparticles or surface modified nanoparticles, and microsphere composite nanoparticles that are larger nanocomposite spheres [64]. 

There are reported examples of the use of these materials in the field of environmental remediation and pollutant detection, as showed in Table 2. 

**Figure 4:**
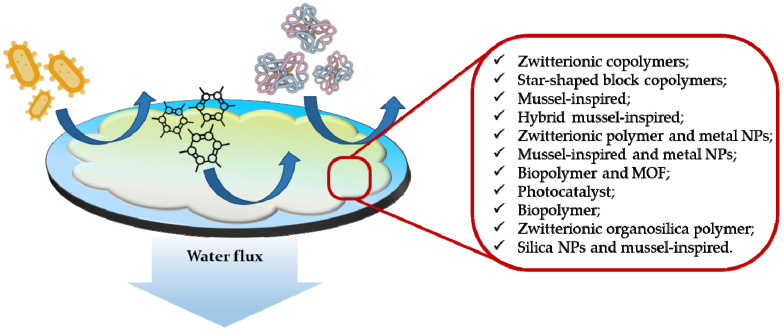
Representation of a general antifouling coated membrane for common water foulants (bacteria, proteins, other organic compounds) and some antifouling functional agents [81].

These functional materials can be combined with already employed remediation techniques using different approaches, such as grafting in polymeric membranes for nanofiltration and ultrafiltration processes to confer on them antifouling properties (Figure 4) [79]. Fouling represents one of the biggest problems of membrane technology, as it compromises filtration efficiency of membranes and reduces their lifespan. It is caused by agents present in wastewater such as inorganic compounds, proteins, bacteria and other organic organisms [80,81].

A poly(vinylidene fluoride) (PVDF) membrane grafted with silver nanoparticles-poly(carboxybetaine methacrylate) (AgNPs-PCBMA) nanocomposites, via a physiosorbed free-radical grafting technique, showed anti-protein and anti-bacterial fouling properties, especially against *E. coli.* In addition, water flux recovery ratio (FRR) and bovine serum albumin (BSA) rejection ratio improved by 40% and 60%, respectively, due to better hydrophilicity performances. These properties were conferred by a synergistic effect due to the presence of AgNPs and the zwitterionic polymer brushes of PCBMA [65].

The catalytic properties of MNPs can also be exploited in the environmental remediation field. For this purpose, a polypropylene hollow fiber membrane functionalized with osmium NPs obtained in situ from a solution of osmium tetroxide in *tert*-butyl alcohol by reduction with molecular hydrogen, was fabricated and employed for a redox process in a membrane contactor. Due to the catalytic properties of OsNPs, the reduction of p-nitrophenol or the oxidation of 10-undecylenic acid was performed with conversions of about 90% and 80%, respectively, in *t*-butanol. The obtained composite membranes have advantages such as large contact reactive area, the ability to operate at relatively low temperatures, to perform in the same module oxidation and reduction processes, no contamination of the working environment, and they represent a useful approach to recover the reaction mass through ultrafiltration [66].

Another example is represented by the use of MNPs for UV/visible-light driven selective oxidation and reduction of some pollutants. Some nanocomposites of Au/Bi_2_WO_6_, fabricated via a hydrothermal approach combined with a rapid reduction–deposition method employing different weight ratios of Au, can be used as visible-light selective photocatalysts in water. In particular, this hybrid nanostructure shows a high capacity for benzylic alcohols oxidation and Cr(VI) reduction in water under visible-light radiation and aerobic conditions. The optimal catalysts for this oxidation/reduction process are 2.0 wt% and 1.0 wt% Au/Bi_2_WO_6_, respectively. The first target of this work was to find a catalyst for selective redox processes in water under the framework of green chemistry, but it also represents a possibility for environmental remediation [67].

Moreover, the oxidation of polluting organic compounds, such as methylene blue, is possible by the employing olfactive photocatalysts in water. For example, a heterostructure based on a plasmonic bimetallic photocatalyst made of Pd-AgNPs/macro porous silicon (macroPSi) can be used to improve the activity of the methylene blue degradation in water under the ultraviolet light illumination. This heterostructure is obtained via a simple immersion process of macroporous silicon for the deposition of monometallic and bimetallic NPs of Ag and Pd. Monometallic photocatalysts AgNPs/macroPSi and PdNPs/macroPSi can also be prepared, but the bimetallic ones showed better performances with a higher efficiency (98.8%) and methylene blue degradation rate (0.033 min^−1^), due to their highest specific surface area and plasmonic effect [68]. 

Other types of aggregates and composite structures that involve MNPs and exploit their catalytic capacities are represented by gels, micelles and vesicles. Microgels based on poly(methacrylic acid-co-acrylonitrile) are obtained by inverse suspension polymerization, and subsequently the nitrile groups are converted into amidoxime groups to obtain a more hydrophilic amidoximated microgel. MNPs based on Cu and Co are synthetized in situ by loading the amid-microgels with the aqueous metal salt solutions of Cu(II) and Co(II) ions, and by subsequent treating with sodium borohydride (NaBH_4_). The obtained microgel composites (amid-p(Mac-co-AN)-M, M: Cu, Co) showed high catalytic effectiveness for the simultaneous degradation of nitrophenoles and cationic and anionic organic dyes (eosin Y, methylene blue and methyl orange), that may exist in contaminated aquatic environments. The ability of such systems to be reused for more catalytic cycles is also observed. In particular, amid-p(Mac-co-AN)-Cu composites can be used up to four times as sacrificial catalyst systems and amid-p(Mac-co-AN)-Co composites do not show any loss in catalytic activity for up to seven cycles, so they appear to be more stable compared to Cu composites in similar aquatic environments. These experimental findings result from the strong coordinating interaction of Co nanoparticles with amidoxime groups [69].

The reduction reaction of 4-nitrophenol can also be facilitated by a pH-responsive multifunctional homopolymer vesicle based on poly[2-hydroxy-3-(naphthalen- 1-ylamino) propyl methacrylate] (PHNA), in which AuNPs are supported. This system showed a synergistic effect between the AuNPs and the supporter (PHNA vesicle). The π-π interaction between the naphthalene pendants in PHNA vesicle occurs with polycyclic aromatic hydrocarbons (less than 0.876 ppb within 1 h) and makes those homopolymer vesicles useful as powerful adsorbents of these contaminants in polluted aquatic environments. This pH-responsive absorbent system (PHNA vesicle) decorated with AuNPs is also recyclable, as it acts as a nanoreactor for the reduction of 4-nitrophenol in water by adding NaBH_4_ [70].

It is also possible to find examples of nanotechnologies based on nanocomposites that combine the antibacterial and catalytic properties of some MNPs and the mechanical properties of natural polymers such as cellulose. A composite material based on cellulose and AgNPs is simple to prepare. By a simple impregnation of cellulose, obtained from citrus waste, with AgNPs, a composite nanomaterial is developed that has antibacterial, antioxidant and photodegradation properties. In particular, discs made of the composite material, cellulose-AgNPs, display more than 90% reduction of *Staphylococcus aureus* culture within 150 min, a moderate total antioxidant potential, minor 2,2-diphenyl 1-picryl-hydrazyl (DPPH) radical scavenging activity, and a moderate photodegradation capacity under sunlight of methylene blue dye of up to 63.16% (time of 60 min) [71]. 

The use of MNPs composites and hybrids is not limited to e environmental remediation and extraction/degradation of pollutants, but is also of high importance for the production of selective sensors for some substances. An example of a detector based on MNPs is a polyurethane micelle/Ag nanoparticle (AgNPs) cluster fabricated as a surface-enhanced Raman scattering (SERS) substrate for in situ extraction and measurement of pesticide residues such as thiabendazole, phosmet, and acetamiprid. Due to their amphiphilic properties, polyurethane micelles have a two-fold function in capturing target molecules and stabilizing the nanoparticle cluster. This method consists of simply dropping the polyurethane micelle/AgNPs substrate on the sample surface (it was tested on surface of apple, orange, and spinach) to extract and detect the target molecules without any previous sample treatment [75]. 

Another example is represented by a reduced graphene oxide detector decorated with AuNPs (rGO@Au NPs), produced to detect heavy metal contaminants in water bodies. In particular, this sensor is able to detect heavy metals such as Cd^2+^, Pb^2+^, Cu^2+^ and Hg^2+^, with a sensitivity of 19.05, 47.76, 22.10 and 29.28 µA µM^−1^cm^−2^, respectively. The rGO@AuNPs are synthetized by a green method utilizing *Abelmoschus esculentus* vegetable extract as a reducing agent, and contaminated water is remediated by the bacteria *Pseudomonas aeruginosa*, *Rhizobium gallicum*, *Staphylococcus aureus*, and *Bacillus subtilis*, that have impressive absorption properties for heavy metal contaminants when acting as scavengers. The cell walls of these bacteria have the tendency to adsorb various inorganic and toxic heavy metal ions, which makes it possible for them to be used successfully for bioremediation. This represents a new green innovative method of detection and bioremediation that can be applied in contaminated aqueous sites and could encourage the development of further eco-friendly technologies for water remediation [76]. 

## 3. Metal Oxide Nanoparticles 

### 3.1. Properties of Metal Oxide NPs

Metal Oxide NPs (MONPs) have intrinsic sorption/desorption, redox, acid–base, photocatalytic and magnetic properties widely used in the environmental remediation field [82,83]. These properties, in combination with polymeric supports, improve the mechanical strength, durability and hydraulic properties of the latter in filtration systems. There are different classes of MONP composites, such as polymer-supported NPs dispersed in polymeric matrices, e.g., for the removal of heavy metals in contaminated water and, also of great importance, magnetically active polymeric particles that have, in both cases, the potential to be reused [84]. 

Tin dioxide, titanium dioxide, zirconium oxide, zinc oxide, cerium oxide, ferric oxide and their nanocomposites with natural polymers, play a key role in the immobilization of active enzymes exploited for bioremediation processes, as these enzymes act in degradation and detoxification of hazardous and carcinogenic compounds. These NPs promote the electron transfer reaction between the active sites on the enzyme and the substrate. The use of MONPs in bioremediation can also enhance the oxidation of hydrocarbons to decrease their toxic effects and support microbial growth [85].

In addition, the magnetic properties of some MONPs, such as Fe oxides NPs, are widely exploited for the recovery of nanocomposite materials based on different organic, inorganic and polymeric matrices, after their use for various remediation purposes such as oil/water separation and removal of dangerous organic and inorganic substances. Various synthesis strategies of iron oxide NPs are described in the literature, such as the co-precipitation method, thermal decomposition, micro-emulsion and hydrothermal/solvothermal synthesis [86].

### 3.2. Metal Oxide NP Composites and Hybrids Applications

The absorption of pollutants from MONP composites occurs through various mechanisms such as complexation, electrostatic attraction, and ion-exchange. Such properties are exploited by the different MONP-based systems listed in Table 3 and described subsequently. 

An example of adsorption based on the formation of inner-sphere surface complexes is represented by a novel nanostructured ternary Fe-Ti-Mn composite oxide. The synthesis used for the development of this composite material consists of a one-step simultaneous oxidation and co-precipitation method. Fe-Ti-Mn (FTMO) composite oxide has a high efficiency for absorption of As(V) and As(III) (especially the latter) by the formation of inner sphere surface complexes under darkness and light at the water/oxide interface. This material is also able to trigger the photo-oxidation and oxidation processes of As(III), due largely to the Ti content in the FTMO adsorbent that under visible or UV light produces electron-hole pairs and a series of reactive oxygen species (ROS) that oxidize the adsorbed As(III) to As(V) at the solid/liquid interface. Mn also contributes to the oxidation process, while the Fe content plays an important role in absorption of the oxidated As(V) formed [87].

Photo-oxidation processes are not the only ones studied in the environmental field; thermochemical processes also play a role as in the conversion Cr(VI) to Cr(III). A composite based on ZnO@TiCN nanourchin, that consists of a heterostructure in which TiCN nanourchins are metallurgically connected to ZnO, synthesized via a solid–solid separation process followed by a wet deposition method, showed the ability to thermochemically convert the adsorbed Cr(VI) to Cr(III) for the complete removal of Cr(VI). Therefore, an adsorption retention of >90% was observed over four regeneration cycles by the ZnO@TiCN nanourchin composite [88].

In wastewater, or in water contaminated by heavy metals in general, it is necessary to act in the removal not only of toxic pollutants such as As(III) and Cr(VI), but also for other ionic metal species such as Cu(II) and Ni(II). For example, the removal of Cr(III), Cu(II) and Ni(II) is exploited by a dendrimer/titania nanocomposite due to a chelation process. This material consists of 4-polyamidoamine (PAMAM) dendrimers with ethylenediamine cores (G4-OH) immobilized on titania (TiO_2_). The simultaneous removal efficiency observed for Cu(II) and Ni(II) ions (with a dosage of 1 g/L) is 99.6% and 99.2%, respectively, after 1 h, while the complete removal of the three metals ions is achieved after 30 min (at pH ≥ 7 for Cu(II) and Cr(III), and pH 9 for Ni(II) ions); therefore, for the mixed metal ion solution an increase in removal efficiency and decrease in the time are observed [89].

Another example of a system that represents a possible solution to the technical limitations of direct NP use in large-scale water treatment and decontamination is represented by composites based on porous granular materials showing properties such as excellent hydraulic characteristics. In this regard, graphene oxide (GO), a layered nonconductive hydrophilic carbon material with a very high density of charged oxygen-containing groups (epoxides, alcohols, ketone carbonyls, and carboxylic groups) [102], is an ideal support for the metal [103] or MONPs. On the other hand, Mn oxide NPs show a high retention capacity for heavy metals, a negative surface charge, a broad pH range, and promote, in combination with GO, the formation of a large porous composite. Therefore, the negative surface of GO promotes the folding of GO nanosheets due to electrostatic repulsion between negatively charged deprotonated oxygen-containing groups of GO at high pH values, and subsequently the formation of a porous composite material with high hydraulic conductivity and low diffusion restriction. An example is given by an Mn oxide NP-impregnated graphene oxide aggregate (GO-MO) nanocomposite, tested by the use of samples containing Cd(II) and Cu(II) as representative metals. Due to an inner-sphere complexation mechanism, the GO-MO composite is able to absorb Cd(II) and Cu(II) with an extremely high uptake rate (> 99.9%) in column operation. The exhausted material can be regenerated by flushing with a 10 BV acid-salt binary solution composed of 0.2 M HCl and 4 wt% CaCl_2_ for the desorption of more than 97% of the preloaded Cu(II) and Cd(II) [90].

Magnetic particles of Fe_3_O_4_ can also be used in combination with GO. An example is shown by Fe_3_O_4_ NPs decorated with graphene oxide and carboxymethylcellulose (Fe_3_O_4_@GOCMC) core-shell structured composite beads, synthesized by a one-pot synthesis and used for the remediation of phosphate and nitrate ions from an aqueous medium. A 0.1 N NaOH solution is used to regenerate the nanocomposite material for the removal of the adsorbed anions. The regenerated Fe_3_O_4_@GOCMC is dried and used for subsequent adsorption-desorption cycles (up to four cycles) [91].

Other porous and more eco-friendly materials, also of plant origin, can be used as absorbents and matrices for MONPs, such as biochar (BC). Biochar is a carbonaceous material produced by pyrolysis, at low temperature and low oxygen conditions, of biomasses such as corncob waste. Due to its large surface area and porous structure, it is used as a soil conditioner in agriculture. It also has a high carbon content and cation exchange capacity and is used, therefore, in carbon sequestration, organic solid waste composting, decontamination of water and wastewater, as a catalyst and activator, electrode materials and as an electrode modifier [104].

Biochar, in combination with NPs (and sometimes stabilizers such as carboxymethyl cellulose) such as Fe NPs for the production of nanocomposites (nano zero-valent iron (nZVI)/BC, iron sulfide/BC, and iron oxide/BC), can be applied in the remediation of heavy metals and organic compounds in the environment. Fe and Fe oxide NP properties improve BC characteristics with a larger surface area, an increased electron transfer efficiency and abundant functional groups, due to their redox, catalytic and magnetic properties. There are different methods for the preparation of Fe/BC composites, including pyrolysis, for obtaining materials that have good ability for contaminant extraction from aqueous solutions and soils, hydrothermal carbonization that uses relatively low temperatures and has fewer disadvantages of direct pyrolysis technology, ball milling with low energy consumption, and fractional precipitation (Fe oxide precipitation and the liquid phase reduction method) with a first step of pristine BC preparation under normal pyrolysis temperature, and a second step of Fe precipitation on the surface and in the inner space of the BC. The remediation mechanisms involving these composite materials include absorption (metals, radionuclides, oxyanions, and organic compounds), reduction (organic compounds and heavy metals) and advanced oxidation processes (of organic dyes, nitroresorcinol, bisphenol A, tetracycline exc.) [105].

Biochar/Fe oxide nano-composites, due to the biochar high absorption capacity and Fe NPs catalytic, adsorption and magnetic separation properties, can be loaded with photosynthetic bacteria (PSB) to produce an eco-friendly and regenerable solution for contaminated wastewater, which can be recovered simply by the use of a magnet bar. A PSB/Fe_3_O_4_/biochar composite showed PSB-supported (*Rhodobacter capsulatus*) enhanced nutrient removal and degradation capability from wastewater. In particular, the removal efficiencies were 83.1% for chemical oxygen demand, 87.5% for NH_4_^+^, and 92.1% for PO_4_^3-^, which remained effective even after five cycles of recycling. Fe_3_O_4_ NPs and Fe oxides generally promote the growth, metabolism and enzyme activities of bacteria and also of PSB, as they act as extracellular electron acceptors to efficiently scavenge reducing equivalents [92].

**Figure 5:**
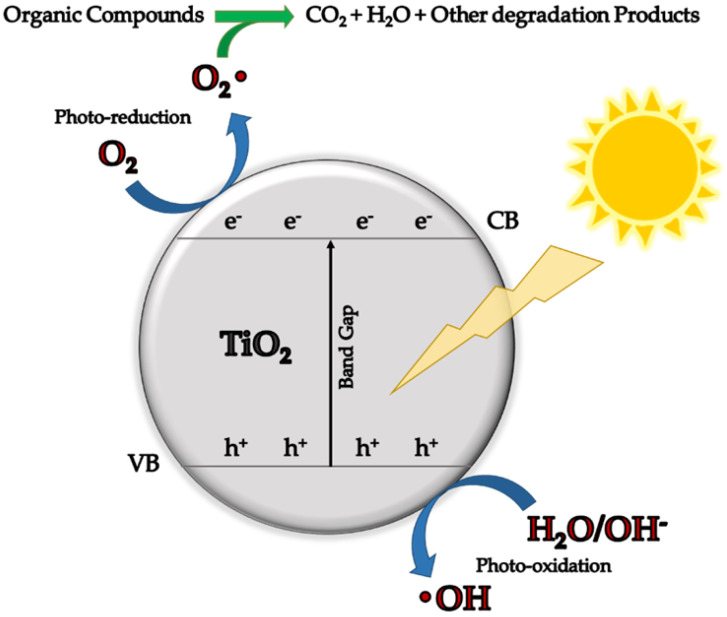
Mechanism of photodegradation of organic compounds by TiO_2_.

Another important metal oxide is titanium dioxide, widely known for its important photocatalytic properties. For this purpose, it is largely used for the synthesis of nanocomposite and nanohybrid materials intended for the environment remediation field and the degradation of different contaminants (Figure 5).

A magnetic TiO_2_ hybridized with lignocellulosic biomass (from olive pits, OP), was synthetized by ultrasonic-assisted sol-gel process and then via hydrothermal magnetization at 180 °C to obtain TiO_2_-OP@Fe_3_O_4_. It was used to remove different dyes (Rhodamine B, Methylene blue and Congo Red) and Cr(VI) from contaminated aquatic systems by absorption and photocatalytic processes under visible light. In particular, the TiO_2_-OP@Fe_3_O_4_ (0.25 g/L) composite showed oxidation rates of 100, 75 and 81% for Rhodamine B, Methylene blue and Congo Red (10 ppm concentration), respectively, and reduction rates of Cr(VI) (10 ppm, pH 3) of 100% (within 40 min). In this case the lignocellulose biomass serves as a matrix for TiO_2_ and Fe_3_O_4_ and to isolate direct contact between them. The presence of the matrix is important because it prevents the dispersion of NPs in treated water and also an unfavorable heterojunction due to increase of the electron/hole charge recombination of pure TiO_2_ and Fe_3_O_4_, which may cause lower photocatalytic performances [93].

Another polymer of natural origin used as a matrix to trap NPs is alginate. By a reverse co-precipitation method, magnetic NPs of Fe_3_O_4_ are synthesized to be encapsulated into alginate and produce alginate magnetic NP beads for the removal of textile blue dyes from water, with a possible use for nano-bioremediation. Different composite beads, based on sodium alginate are obtained by the use of different types of magnetite NPs. Therefore, a first type of bead is prepared with ferrous sulphate, a second one with ferrous sulphate and biochar, and a third one with ferrous sulphate, biochar and graphite. An 82.4% removal of azo blue dye (25 ppm) was observed after 3 h of equilibrium time, and 55.22% at pH of 8 for the initial dye concentration of 100 ppm, by the ferrous sulphate, biochar and graphite alginate bead [96].

Polymer and NP-based nanocomposites are also used for oil spill remediation and recovery. Magnetic shell cross-linked knedel-like nanoparticles (MSCKs) with amphiphilic organic domains and a magnetic sensitive core, are obtained through the co-assembly of amphiphilic block copolymers of poly(acrylicacid)-block-polystyrene (PAA20-b-PS280) and oleic acid-stabilized magnetic iron oxide NPs, and are used for the adsorption of hydrophobic guest molecules. These guest molecules are synthetized by a thermal decomposition method. The co-assembly of amphiphilic block copolymers of PAA20-b-PS280 and hydrophobic magnetic nanoparticles is performed using tetrahydrofuran, N,N-dimethylformamide, and water, while the cross-linking of the obtained hybrid micelles is achieved by an amidation process. The MSCK nanocomposites have been used for a complex crude oil sorption test in water, with an oil sorption capacity of 10-fold the initial dry weight. After their use, they are regenerated by washing in ethanol with the help of sonication [97].

By using appropriate bacteria, biosurfactants and remediation enzymes, it is possible to bioremediate oil from contaminated aqueous sites [106,107]. A bionanocompound consists of polyether amine (PEA) coated magnetite NPs, was obtained to immobilize *OmpA* (a biosurfactant) and Laccase (a remediation enzyme) for the demulsification of oil/water emulsions, and removal/degradation of oils. Magnetite NPs were synthesized by a co-precipitation method before activation by silanization with (3-Aminopropyl)triethoxysilane (APTES) and modification with glutaraldehyde to conjugate the obtained Magnetite NP-AP-GA with oxidazed polyether amine (PEA). To obtain Magnetite NP-PEA-OmpA-Laccase bionanocompounds, the surfactant OmpA and the enzyme laccase were conjugated with Magnetite NP-PEA. The bionanocompounds Magnetite NP-PEA-OmpA and Magnetite NP-PEA-OmpA-Laccase at 5000 ppm achieved the separation of a 1 wt% oil/water emulsion, also showing a heavy crude oil removal efficiency, by applying an external magnetic field of 81% and 88%, respectively, and degradation levels between 5% and 50% [98]. 

## 4. Carbon-Based Nanomaterials 

### 4.1. Properties of Carbon-Based Nanomaterials

Due to their singular chemical and physical properties, such as excellent electrical and heat conductivity, advanced optical properties, chemical stability and high mechanical strength, carbon-based nanomaterials have attracted a lot of interest from the scientific community. They consist of solid-state carbon allotropes composed of sp^3^ and/or sp^2^ hybridized carbon atoms with different nanoscale dimensions, such as zero-dimensional (0D) fullerenes, nanodiamonds and graphene quantum dots, one-dimensional (1D) single walled or multiwalled carbon nanotubes (SWCNTs and MWCNTs) and two-dimensional (2D) graphene and graphene oxide. These nanomaterials can be obtained by different methods such as (i) ‘‘bottom-up’’ approaches, such as chemical vapor deposition (CVD) on a substrate such for SWCNTs, MWCNTs and graphene; (ii) synthesis approaches from aromatic benzene derivatives, and (iii) ‘‘top-down’’ methods, such as sonication liquid phase exfoliation, and ionic-liquid assisted electrochemical exfoliation for graphene [108]. Carbon-based nanomaterials have applications in various sectors, such as the biomedical sector, due to their antibacterial properties [109], theranostic and tissue and cell imaging based on their one-photon and two-photon fluorescence properties [110], energy storage and conversion, catalysis [111], material design and synthesis for environmental remediation for the removal of organic molecules, heavy metals and oil/water separation. There have been several studies for the application of these nanomaterials in the field of environmental remediation, in particular of their composites to improve water contaminants absorption capacity and make them regenerable. For these latter purposes, porous graphene materials, such as foams, sponges and aerogels, that have improved mechanical, physical and chemical (hydrophilic-lipophilic) properties, have been produced [112]. The cellular toxicity of some carbon-based nanomaterials [113] has been studied, demonstrating their evident size-dependent hazards and cytotoxicity enhanced, in particular, after their surface functionalization with acid treatments, due to the presence in those materials of carbonyl, carboxyl, and/or hydroxyl groups [114]. Therefore, it is even more important for safe-by-design [115] composite functional materials that incorporate these nanomaterials to avoid their dispersion in the environment.

### 4.2. Carbon-Based Nanomaterial Composites and Hybrids Applications

Some applications of carbon-based nanomaterial composites and hybrids in the field of remediation and bioremediation of different pollutants, are listed in Table 4. 

A field of remediation of contaminated aquatic sites concerns the separation of oil/water mixtures. For this purpose, there are many approaches used that exploit nanocomposites, modulating their hydrophilic/hydrophobic properties through appropriate chemical functionalization. With carbon-based nanomaterials, beyond hydrophobic interactions it is also possible to exploit π-π interactions, hydrogen bonds, and electrostatic interactions for the trapping of water pollutants. Adsorbent materials are thereby developed to remediate contaminants such as oil spilled on water. Nanosponges are a category of these porous materials. In particular, an example is represented by a hydrophobic floatable absorbent, obtained by a chemical vapor deposition (CVD) approach, made of carbon nanotubes (CNTs) and nanofibers grown up on the surface of a vermiculite (a clay mineral) expanded substrate. The absorption of different oils has been studied, with an increase in absorption capacity of ca. 600% and a decrease of undesirable water absorption, due to the hydrophobicity of the composite material with respect to the original vermiculite [116].

**Figure 6:**
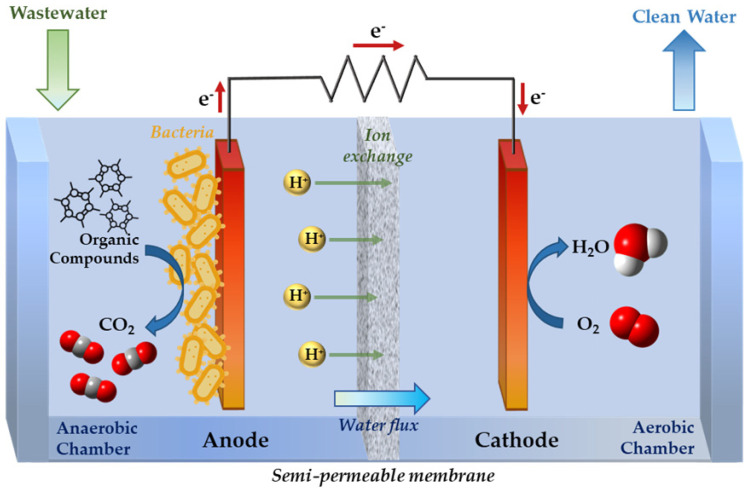
Schematic representation of a microbial fuel cell for the degradation of organic pollutants.

CNTs can be also used for in situ and ex situ bioremediation processes. They show the capacity to increase the growth of some microbial communities in oil-contaminated freshwater sediments to promote the in situ degradation of these contaminants [131]. Due to their electrical conductivity, biocompatibility, lack of corrosiveness, and chemical stability, they can also be used in combination with conducting polymers such as polyaniline (PANI) and polypyrrole (Ppy) for electrode modification in microbial fuel cells (MFCs) for bio-electrochemical ex situ treatments. Microbial fuel cells are an emerging and promising technology able to convert chemical energy into electrical energy in the presence of a biocatalyst (electroactive bacteria species) and simultaneously remove toxic pollutants such as organic dyes, COD and phenol from water (Figure 6), in combination with nanomaterials such as carbon-based NPs and conductive polymers, as electrodes. Electrodes, in particular anodes in MFCs, have the function of collecting electrons produced by the biodegradation processes of pollutants, and producing energy [132]. 

In this regard, a polyaniline/carbon nanotube (PANi/CNT) hybrid composite was prepared by an in situ oxidative chemical polymerization method for the generation of bioelectricity from wastewater and its treatment. In particular, the PANi/CNT composite was used for the preparation of an anode electrode for MFCs. This system is characterized by the presence of π-π interactions between PANi and CNT, showing a strong interaction between the microbe and electrode on biofilm attachment. Due to its charge-transfer capacity, this composite is able to generate bioelectricity and, at the same time, perform 80% COD removal from wastewater [86].

Another use of MFCs with CNT composites is represented by bare and multiwalled carbon nanotube/polypyrrole (MWCNT/Ppy) coated electrodes used for bioelectrochemical treatment of phenol in anodic and cathodic compartments under aerobic and anaerobic conditions. The best results are obtained for the biocathodic treatment and aerobic conditions with high removal efficiencies of phenol and COD [118].

Bioremediation of azo dyes and Cr(VI) can be performed by the use of metal oxide NPs, as described in the previous paragraphs, and also by their combination with CNTs. For this purpose, anthraquinone-2-sulfonic acid or humic acids and Fe_3_O_4_ are immobilized on CNTs (AQS/Fe_3_O_4_/CNTs and HA/Fe_3_O_4_/CNTs) for the reduction of Cr(VI) and methyl orange from wastewater. This process is exploited by capturing electrons from the microbial metabolism of anaerobic bacteria, with a high effectiveness at pH 8.0 and excellent stability and reusability. Dissolved organic matter (DOM) and Fe(III) on carbon nanotubes, still have electron transfer capacity, and the dispersion of residual DOM and Fe after the bioreduction of pollutants is prevented by their immobilization in CNTs. Reusability of this material is possible due to its magnetic properties by use of a magnet bar after the absorption of azo dyes and Cr(VI). It can be recovered and regenerated by washing with ethanol and deionized water and drying [119].

If CNT composites enable the development of remediation technologies of environmental pollutants such as heavy metals, organic dyes and other organic substances such as hydrocarbons, due to their absorption, photocatalytic and electrocatalytic properties, on the other hand there are various examples of the use in this sector of other carbon-based nanomaterial, i.e., graphene and its derivative graphene oxide (GO). GO is a layered nonconductive hydrophilic carbon material produced by the oxidation and exfoliation of graphite by oxidant agents (Hummer’s method KMnO_4_, NaNO_3_, H_2_SO_4_ [133]), and is characterized by a very high density of charged oxygen-containing groups (epoxides, alcohols, ketone carbonyls, and carboxylic groups). GO shows high adsorption capacity of different molecules via physical and/or chemical forces, such as electrostatic, π-π and hydrophobic interactions, due to its negatively charged surface. There are two main approaches for the functionalization of GO and obtaining nanocomposites: the use of GO as a host material, and the use of GO in host materials. In the first case, the functionalization of GO surface with various approaches is performed. In the second, crosslinking between GO and a polymeric matrix is conducted by following a simple and non-toxic synthesis to obtain a nanocomposite with a stable structural configuration, high absorption capacity with easy recovery and regeneration after the use [134]. 

Composite aerogels are porous materials that have applications in the water remediation field due to their adsorption properties. In particular, aerogels with high hydrophobicity that float in aquatic environments can be produced for easy use and recovery. An example is represented by a novel graphene aerogel/Fe_3_O_4_/polystyrene composite produced by an environmentally friendly and cost-effective solvothermal technique. This aerogel is characterized by the presence of porous Fe_3_O_4_ NPs that act as cross-linkers for graphene oxide plates (produced by a modified Hummer’s method), and polystyrene that allows with Fe_3_O_4_ NPs the formation of a porous structure with enhanced hydrophobicity of the composite aerogel. The ultralow density aerogel composite has a crude oil absorption capacity of 40 times its own mass after 10 water-oil separation cycles, and is easily recovered due its floating capacity. It also has magnetic properties, due to the presence of Fe_3_O_4_ NPs, that permits collection of the exhausted aerogel with a magnet. The regeneration of the aerogel is performed by simple squeezing [120].

GO, as well as CNTs, can also be used in combination with innovative bioremediation techniques, such as MFCs, for the preparation of anode electrodes that allow the production of energy and, at the same time, the remediation of pollutants such as heavy metals. This remediation technology is also related to the design and synthesis of new innovative composite materials, also from natural resources. The production of GO starting from organic biomass is an example, and due to its chemical inertness, conductivity, mechanical robustness and flexibility, good specific surface area, and high biocompatibility, may be an ideal candidate for production of a bioinspired anode material for MFCs. A graphene derivative (L-GO) is produced from organic oil palm biomass, first by the extraction of lignin powder, using a soda pulping process. Then, the carbon flakes are obtained by an annealing process and oxidated by Hummer’s method. For the improvement of GO electron transportation in MFCs, ZnO NPs are also synthetized with a green solvothermal method from lemon peel to produce a L-GO/ZnO composite. For extra mechanical strengthening in the preparation of the anode, the L-GO/ZnO electrodes are dipped into a polylactic acid (PLA) solution. The resulting anode is tested for the production of energy and remediation of Pb^2+^ ions from wastewater through MFC, showing excellent energy generation and remediation efficiencies and the presence of bacterial species such as *Lysinibacillus* sp., *Klebsiella* sp. and *Leucobacter* sp. detected on its surface [121].

Sucrose is another natural, highly carbonaceous, organic source useful for the production of graphene derivatives. A graphenized, sand-based composite adsorbent with large porosity and surface area was obtained by an acid and high temperature treatment of sand-coated sucrose, and tested for the absorption of Hg^2+^ from contaminated water with a quite economic and environmentally friendly approach. The absorption capacity of Hg^2+^ (299.40 mg/g) shown by the composite material was high, due to the interaction of the metal ion with the functional groups, as observed with FTIR analysis. The chelated bonds between the Hg^2+^ and the composite material are weakened at acidic pH, so it is possible to regenerate it with a desorption process, thus allowing its reusability [122].

A green solvothermal method can be used for the production of more complex hybrid nanocomposite materials for the remediation of contaminated aquatic sites, such as a nano-sized nickel-benzene dicarboxylate (Ni-BDC) metal organic framework (MOF) decorated over graphene oxide (GO) or a carbon nanotube (CNT) platform. This nanocomposite, in particular Ni-BDC@GO, shows high retention capacity of methylene blue (222 mg/g) due to the presence of π-π interaction and has excellent regeneration. Indeed, all of the nanocomposites can be regenerated by centrifugation and simple washing with alcohol for other absorption cycles [123].

Methylene blue and other organic dyes can be degraded through the photocatalytic action of metal oxide NPs such as TiO_2_, ZnO and Bi_2_O_3_, as already described. In this regard, graphene, a nanomaterial easy obtained from GO by reduction processes, can be exploited for its photocatalytic activity in contaminants removal, and in combination with metal oxide NPs. It was discovered that graphene incorporated with composite nanofibers (TZB-Gr) (titanium dioxide-zinc oxide-bismuth oxide-graphene), prepared by a sol-gel based nozzle-less electrospinning process, due to the coupling of 2D graphene with photoactive semiconductors nanofibers, improved the removal capacity of organic dyes. Under visible-light and UV-light irradiation, the TZB-Gr composite nanofibers can activate organic dyes to produce ·O_2_− and ·OH radicals that are powerful oxidizing species for the degradation of most organic pollutants [124]. 

Another example of a nanocomposite, which combines graphene and metal oxide NP properties, is represented by a TiO_2_-graphene (P25-GR) composite obtained by a hydrothermal reaction of GO with TiO_2_ (P25) with different graphene ratios. This composite material has been tested for the absorption and photodegradation of polycyclic aromatic hydrocarbons (PAHs) (phenanthrene, fluoranthene, and benzo[a]pyrene as models). The P25-GR with a 2.5% of graphene showed the best results for absorption, charge transportation and photocatalytic performance (~80% of PAHs removed after 2 h) at high PAH concentrations (2.0–4.0 µg/mL) in alkaline conditions [125].

The applications of carbon-based nanocomposites made of carbon nanotubes, graphene and graphene oxide, and also fullerene composites, have been studied for their potential applications in the water remediation field. A titania-based composite based on zinc porphyrin covalently functionalized fullerene [C60] derivatives, was prepared for the sensitization of titania as a visible-light active photocatalyst for river and wastewater remediation (phenol and methylene blue as models). This material showed interesting results for its potential applications in the remediation of a series of complex environmental and industrial matrices [126].

## 5. Silica-Based Nanomaterials

### 5.1. Properties of Silica-Based Nanomaterials

Silica NPs can be obtained by a simple synthetic route via the sol-gel process. This is a two steps synthesis that involves the formation of monodisperse silica NPs starting from aqueous alcohol solutions of silicon alkoxides in the presence of ammonia as a catalyst. The sol-gel first step is a hydrolysis reaction for the formation of silanol groups, while the second step is a condensation polymerization reaction to form siloxane bridges [135], as in the following Equations (1) and (2):

Si–(OR)_4_ + H_2_O → Si–(OH)_4_ + 4 R–OH (1)

2 Si–(OH)_4_ → 2 (Si–O–Si) + 4 H_2_O (2)

The sol-gel technique is also widely used for the functionalization and surface modification of different materials with the aim of producing advanced and multifunctional materials with properties such as UV radiation protection, antimicrobial finishing, water repellency, bio-molecule or functional-molecule immobilization, flame retardancy, chemical resistance and self-cleaning properties [136,137,138,139,140,141].

However, porous silica nanomaterials have better performance in various sectors due to their large surface area and the presence of large pores that act as hosts for different molecules. Two categories of porous silica materials are silicate or aluminosilicate (minerals composed of aluminium, silicon, and oxygen, plus counteractions) based on nanomaterials such as zeolites and mesoporous silica NPs with well-defined pore networks. 

Mesoporous silica NPs are highly versatile nanomaterials obtained from silicate gels and quaternary ammonium surfactants of different chain lengths which, due to their high surface area, large pore volumes, tunable pore size and easy surface modification properties, have applications in various sectors such as drug delivery, nanomedicine, theranostics and photodynamic therapy [142]. Due also to the absorption properties of mesoporous silica NPs and their composites, they have applications in environmental remediation for the adsorption of various contaminants from aqueous or gaseous media [143,144]. 

Zeolites are crystalline aluminosilicates (or silicates), commonly produced with hydrothermal conditions by the use of organic templates, with a regular spatial arrangement of uniform cages, cavities or channels of molecular dimensions, high surface area, and cation exchange properties. Zeolites have applications in various sectors such as catalysis and renewable energy applications [145], but also in the absorption and retention of environmental contaminants, due to their high selectivity for inorganic cations (zinc, lead, cadmium, copper, nickel), anions, and organic compounds such as pesticides and phenols [146].

**Figure 7:**
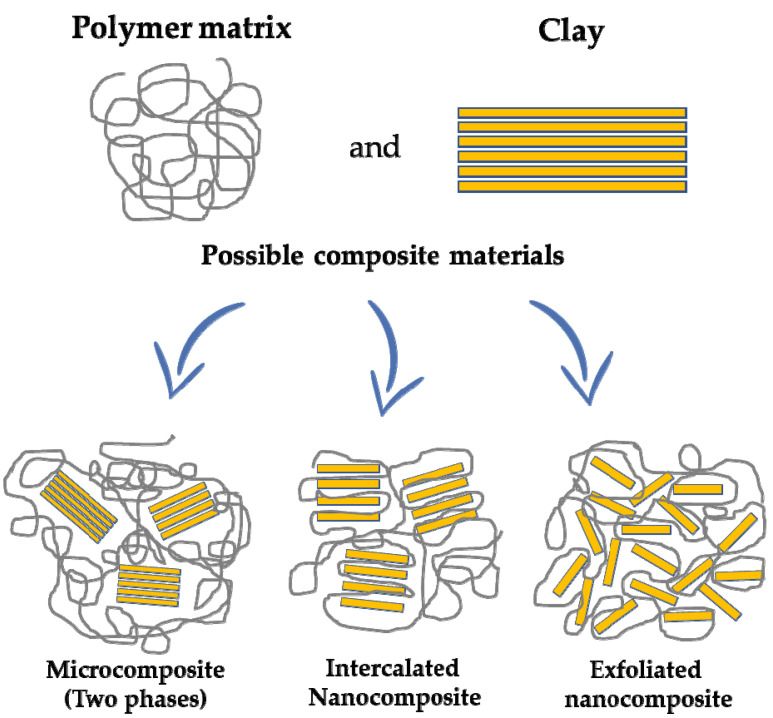
Different types of clay-based composite materials.

Other minerals based on aluminosilicates are clays, defined as nanostructured fine-grained minerals. They can be classified into different groups such as montmorillonite, kaolinite, illite, bentonite and chlorite, due to the different mineral composition, size and structure. Clays are characterized by high surface area, swelling capacity (in particular the classes of montmorillonite and bentonite clays), cation exchange capacity and strong adsorption/absorption properties due to their negatively charged surface [147]. They are also easily functionalized by modification of their hydrophilicity/hydrophobicity surface and adsorption properties, to obtain functional clays or composites (Figure 7) [148,149,150] with applications in the environmental remediation field [151,152].

### 5.2. Silica-Based Nanomaterial Composites and Hybrid Applications

Silica-based nanomaterial composites and hybrids have great potential for environmental remediation and bioremediation. Some examples are reported in Table 5. 

In this regard, an inorganic–organic hybrid absorbent, based on polypyrrole/hollow mesoporous silica particles (Ppy/HMSNs), and obtained by in-situ polymerization, showed (in particular the nanohybrid with pyrrole content of 60 wt %) a high and selective adsorption and removal rate (of 100%) of Cr(VI) from contaminated water with a maximum absorption of 322 mg/g at 25 °C, strong reduction ability of Cr(VI) to the lower toxic Cr(III), and high reusability, with a removal rate of Cr(VI) after five cycles of adsorption–desorption still more than 97% [153].

Mesoporous silica can also be configured as nanotubes, leading to the preparation of new nanocomposites with metal NPs able to perform catalytic processes for water decontamination. PdNPs supported on hollow mesoporous silica nanotubes (h-mSiO_2_), as prepared by a sacrificial template and impregnation-reduction method, can be used for the catalytic reduction of 4-nitrophenol and hydrodechlorination of 4-chlorophenol in wastewater. Mesoporous silica nanotubes can be obtained through a biphase stratification approach by wrapping the silica shell on MWCNTs or Pd/MWNTs, and later by calcination to remove the MWNTs. By this method, Pd@h-mSiO_2_ and h-mSiO_2_ are obtained. These two nanocomposites, in which Pd is uniformly assembled in the exterior and interior of the mesoporous silica nanotube, have excellent catalytic activity for the reduction of 4-nitrophenol and for the hydrodechlorination of 4-chlorophenol, even after six cycles of utilization, due to the enhancement of the Pd active site accessibility and mass transfer of the reactants [154]. 

Mesoporous silica composites also have a great absorption response for emerging pollutants such as antibiotics. A composite nanomaterial based on mesoporous silica NPs and magnetic GO, as obtained by the modification of GO with Fe_3_O_4_ NPs by a hydrothermal method, was prepared and utilized to remove a sulfamethoxazole antibiotic, with a maximum absorption of 15.46 mg/g (fivefold superior adsorption capacity of the pristine magnetic GO), from environmental water samples. This was due to different adsorption mechanisms such as hydrogen bonding, electrostatic interactions and π-π interactions [155]. 

Bioremediation processes can also be exploited by magnetic composites based on mesoporous silica NPs through the immobilization of enzymes such as laccase. An example is represented by mesoporous silica-coated magnetic multiwalled carbon nanotubes (Fe_3_O_4_-MWCNTs@SiO_2_) used for the immobilization of the laccase enzyme with glutaraldehyde as a cross-linker, to serve as a biocatalyst for the discoloration of synthetic organic dyes. In particular, this biocatalyst has been tested for the discoloration of Eriochrome Black T, Acid Red 88, and Reactive Black 5, without a mediator, showing a decolorization efficiency up to 99, 98 and 66%, respectively, and also for the oxidation of 2,2′-azino-bis(3-ethylbenzothiazoline-6-sulfonic acid), retaining 87% of its original activity even after 10 cycles of use [156].

Laccase and mesoporous silica composites can also be used for the bioremediation and oxidation of phytotoxic phenolic compounds present in olive mill wastewaters. In this regard, a biocatalyst was prepared by the immobilization of laccase from Pleurotus sajor-caju on SBA-15 mesoporous silica functionalized with 3-aminopropyltrimethoxysilane and activated with glutaraldehyde. The obtained nanobiocomposite had high stability. Indeed, it was stable in aqueous solution for more than 10 reaction cycles of oxidation of four phenolic compounds tested (protocatechuic acid, ferulic acid, sinapic acid and caffeic acid present in olive mill wastewaters), with a conversion of 84 mol% [157].

Zeolite composites have different applications in the environmental remediation sector, and the use of these composites in bioremediation processes has also been reported. In particular, the properties of zeolites can be implemented by their combination with other nanomaterials, such as metal or metal oxide NPs, to obtain nanocomposites with magnetic, catalytic or photocatalytic properties. 

An example is represented by magnetic graphene oxide modified zeolite (Cu-Z-GO-M) composites obtained by a solid-state dispersion method. Different composites with different GO/Cu-zeolite ratios have been synthetized to perform adsorption tests using methylene blue as a representative contaminant. The best adsorption results were achieved by composites with a higher GO ratio at pH > 9, because this enhances the removal ability of zeolite due to the introduction of active sites and an increased surface area. Higher temperature could improve the removal ability of methylene blue, that for the Cu-Z-GO-M 1:1 composite resulted in 82.147, 89.315, 97.346 mg/g at 298, 308, and 318 K, respectively. The nanocomposite can be easily recovered by a magnet, due to its magnetic properties, and regenerated with 0.1 mol/L HCl solution as the strippant, with a removal efficiency of methylene blue of 76.86% (for Cu-Z-GO-M 1:1) after five cycles with reuse [159].

Zeolites from waste or natural resources are a low-cost solution when compared to artificial zeolites. A bimetallic composite of a fly ash-based zeolite supported with nano zerovalent iron and nickel (nZVI/Ni@FZA) combines the absorption properties of zeolite and the catalytic properties of metal NPs. This material can be used to perform the potential removal of Cr(VI) and Cu(II) contaminants from polluted aquatic sites by reduction, adsorption, and ion exchange mechanisms. In particular, this composite showed a maximum adsorption capacity of 48.31 mg/g and 147.06 mg/g towards Cr(VI) and Cu(II), respectively. It has also been demonstrated that the presence of Ni in the bimetallic nanocomposite enhances the reduction properties of nano zerovalent Fe [160].

Clinoptilolite, a natural zeolite, can also act as a support for TiO_2_ to obtain a composite with photocatalytic properties. A TiO_2_-based Mexican natural zeolite composite (T/MZ) was studied for the photodegradation of metoprolol, an emerging contaminant used as beta-blocker medicament and employed in the treatment of hypertension, arrhythmia and heart failure. Moreover, it was demonstrated that the zeolite acts not only as a support, but can also enhance the photocatalytic activity of pristine TiO_2_ under near-UV light [161].

Zeolites can also be used for bioremediation processes. For example, a nanobiocatalyst of zeolite and GO prepared by silanization with (3-aminopropyl)trimethoxysilane and crosslinked with glutaraldehyde, was used for the immobilization of laccase enzyme. GO facilitates the electron transfer for the immobilized enzyme active sites. This nano-biocatalyst is used for the degradation of Direct Red 23 as a representative organic pollutant, and regenerated by washing with distilled water, showing good reusability over five cycles, high storage stability, and thermal stability [162]. 

There are also applications of zeolite nanocomposites in soil bioremediation. A bio-polymer composed of Pseudomonas veronii cells grown to stationary phase and immobilized in a xanthan gum-based biopolymer via encapsulation was used as a coating on a natural zeolite to produce a nanocomposite tested for the bioremediation of Hg^2+^ from contaminated soil. The immobilized cells led to Hg reduction and subsequent production of gaseous elemental mercury through a volatilization mechanism. This composite could be also stored for at least four months and transported intercontinentally without any loss of functionality, proving to be a possible solution for the bioregeneration of mercury-contaminated soils [163]. 

Nanocomposites based on different clays (i.e., montmorillonite, kaolinite and halloysite) are also very interesting [171]. Halloysite is a natural tubular clay, and one of the most studied, due to its biocompatibility (with low in vivo and in vitro toxicity) and mechanical, physical and chemical (simple inner/outer surfaces functionalization) properties. Due also to its tubular form, it can be loaded with different species to be used for the release of active molecules such as antioxidants [172], flame-retardants, corrosion inhibitors [173], biocides and drugs [174]. In this regard, there are many examples of the use of halloysite loaded or functionalized with different nanomaterials, such as metal or magnetic NPs, for environmental remediation approaches, that are able either to perform pollutant degradation catalytic reactions or to be used as recoverable absorbent systems. Halloysite can be functionalized with organosilanes by simple reactions such as sol-gel synthesis to improve dispersibility into polymer matrices, to enhance thermal stability and tensile properties, and also to produce hydrophobic nanohybrids for different applications [175].

The inner layer of halloysite can also be hydrophobized with surfactants, such as octadecyl phosphonic acid, to provide more sites for MNPs binding by neutralization of the positive charge present in the inner alumina layer, and for the loading of hydrophobic molecules such as oil and organic pollutants. On the other hand, halloysite with its hydrophobic surface can form stable oil-water emulsions and can encapsulate water droplets inside the clay shell to form liquid marbles that can be used for the encapsulation of biomaterials or biofilm-producing bacterial species, such as the hydrocarbonoclastic bacteria Alcanivorax borkumensis, to provide a mechanical reinforcement for such encapsulation and perform oil-spill bioremediation processes [176]. 

A hydrophobic halloysite obtained by the functionalization with octadecyltrimethylsilazane (ODTMS) was used for pickering emulsion formation with crude oil, showing better bacterial attachment to oil micro-droplets compared to artificial surfactants. The modification of halloysite by an outside clay tube coating and inner-loading with organic surfactant and nutrition-enhancers, can also improve the halloysite stabilization of oil emulsions and Alcanivorax borkumensis proliferation [165]. This modified halloysite, with a hydrophobic surface, can also be used for the coating absorbent porous materials such as foams to obtain a more efficient absorbent technology for organic solvents and oil remediation. An example is represented by a polyurethane foam coated with a polysiloxane-modified halloysite (POS@HNT) to obtain a recyclable composite material with flame-retardant properties and a hydrophobic surface. The modification of the foam is performed by a dip-coating process of the polyurethane foam with hydrophobic halloysite, obtained by the condensation reaction of hexadecyltrimethoxysilane and tetraethoxysilane. The obtained modified polyurethane foam showed a water contact angle greater than 150°, and an absorption ratio for chloroform and dichloroethane of 104 and 74, respectively. It is also recyclable by squeezing, maintaining oil absorption performances even after 10 absorption squeezing cycles [167].

Due to their absorption and mechanical properties, other clays can be used for the preparation of functional nanocomposites applied to bioremediation processes. The use of clays in composite materials, such as membranes, results in a reduction of the toxic effects of some nanomaterials, such as GO nanoplates, due to the aggregation of the clay NPs leading to a weakening of their chemical properties [177].

Furthermore, nanocomposites based on clays and metal oxide NPs that show catalytic properties can be used in combination with macrocycles such as cyclodextrin to improve their affinity and degradation for organic pollutants. An iron-clay-cyclodextrin composite absorbent catalyst was produced by coating montmorillonite clay with iron-oxide and modifying the obtained composite with cyclodextrin monomers and polymers cross-linked with polyfluorinated aromatic molecules. This material showed a high absorption of the model pollutants, bisphenol A (BPA), carbamazepine (CBZ), and perfluorooctanoic acid (PFOA), and excellent degradation efficiency using H_2_O_2_ (over 90% in 1 h for BPA and CBZ and ∼80% for PFOA), remained constant also fand was unchanged after five consecutive cycles of absorption and degradation [168]. 

Catalytic degradation of organic pollutants can be performed with laccase enzyme. A chitosan-clay composite bead was used for the immobilization of laccase from Alcaligenes faecalis XFI strain, showing a higher efficiency in enzyme immobilization than simple chitosan beads due to the synergetic effect of clay (mechanical strength) and chitosan (porous nature) and their better biocompatibility with the laccase enzyme. The chitosan-clay-laccase nanobiocomposite was tested for the degradation of three synthetic dyes, showing maximum decolorization percentage for anthrax quinone dye of 85%, azo dye of 82% and diazodye of 69%, and good reusability [169].

## 6. Final Remarks and Future Perspectives

**Figure 8:**
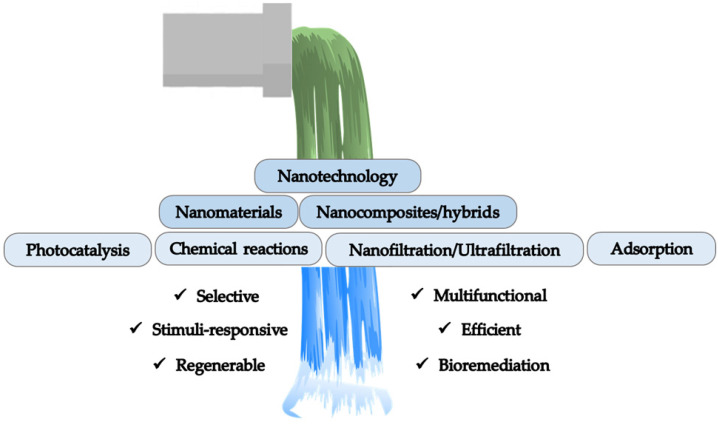
Advantages in the use of nanotechnology for wastewater treatment processes.

In the light of the examples examined in this review, some observation can be made. Nanohybrids and nanocomposites present numerous unique advantages, as shown in Figure 8 and listed below. These include:

- easy preparation by eco-friendly approaches;
- almost unlimited possibilities of functionalization;
- versatility in term of shape and application forms;
- selectivity of adsorption;
- good stability;
- degradation of pollutants performed by catalytic or photocatalytic processes;
- ability to be reused for numerous cycles of adsorption, and regenerated;
- possibility to be exploited for bioremediation processes.

Furthermore, nanohybrids and nanocomposites can have lower toxicity than pristine nanomaterials. An example is represented by carbon-based nanomaterials or metal NPs that, once employed as nanofillers stably incorporated and chemically bonded into polymeric 3D matrices, show good affinity towards biological systems and cell membranes, with no cytotoxicity or dispersion into the environment [178,179]. 

It is worthwhile to remark that most of the mentioned nanotechnological examples for (bio)remediation reported in the literature still have poor scalability for real environment applications.

Furthermore, in some cases, the cost/method of production still does not justify the benefit achieved for their use in (bio)remediation applications, and does not allow their widespread use in the environmental technologies market. In this regard, research needs to move more towards the preparation of nanohybrids and nanocomposites that fit the needs of real-use case applications, with large-scale production, safe use by the final users/experts, and commercially competitive viability regarding cost/benefit ratio. In accordance with circular economic principles, the aim of this review was to address the actual need to design and produce new materials with a lower carbon footprint by following an accurate life cycle assessment to evaluate their impact on the environment.

## 7. Conclusions

In this review the latest achievements in the field of environmental remediation are reported with respect to exploiting the use of nanotechnology to perform more efficient water and soil treatment processes. 

In particular, recent potential solutions for environmental treatment processes are described with respect to nanocomposites and hybrids materials based on four different classes of nanomaterials: metal nanoparticles, metal oxide nanoparticles, carbon-based nanomaterials and silica-based nanomaterials. The composite materials exhibit catalytic (or photocatalytic), redox, sorption/desorption, and magnetic properties, featuring the different nanomaterials used as nanofillers. Many examples are also reported concerning the combination of different nanomaterials for the synthesis of composite functional materials featuring all the properties intrinsic to the starting nanofillers and even new materials. One of the advantages of the described composite materials concerns their regeneration and reuse ability, with low efficiency loss even after several adsorption cycles. Furthermore, their degradation capacity is explained towards some organic pollutants, through catalytic, photocatalytic or electrochemical processes. 

Eco-friendly synthetic processes and approaches of more sustainable materials are reported using bio-based polymers incorporating functional nanomaterials. Most importantly, the possible use of these innovative materials and solutions is underlined with in situ and ex situ bioremediation processes, as in systems such as microbial fuel cells, and by immobilization of bacteria or enzymes for degradation processes. 

This review demonstrates how it is possible to develop, using safe-by-design innovative composites and hybrid materials, new sustainable solutions for the removal of common and emerging pollutants from contaminated water and soils, with a view to the recovery and reuse of such materials, for more efficient, low energy consumption and eco-friendly processes. 

## Figures and Tables

**Table 1:** List of some environmental hazardous substances.

Type of Substance	Pollutant	Ref.
Heavy metal	As, Cd, Cr, Cu, Pb, Hg, Ni, Zn	[23]
Radionuclide	^3^H, ^14^C, ^90^Sr, ^99^Tc, ^129^I, ^137^Cs, ^237^Np, ^241^Am	[24]
Fertilizer	Ammonium nitrate, phosphate	[25]
Monocyclic and bicyclic aromatic hydrocarbon	Benzene, toluene, xylenes, styrene, naphthalene, byphenyl	[26]
Polycyclic aromatic hydrocarbon	Benzo(a)pyrene, benz(a)anthracene, indeno(1,2,3-cd)pyrene	[27]
Halogenated aromatic (and polycyclic) hydrocarbon	Chlorobenzene, dichlorobenzene, 4-chloropyrene, 2-bromofluorene, 2,3,7,8-tetrachlorodibenzo-*p*-dioxin	[28,29]
Nitrogen-containing	Nitrobenzene, caffeine	[30,31]
Phenol	Phenol, 4-nitrophenol, 2-chlorophenol, bisphenol A	[32,33]
Ether	Diphenyl ether, dibenzofuran	[34]
Aliphatic hydrocarbon	*n*-alkanes	[35]
Insecticide	Acetamiprid, deltamethrin, endosulfan, malathion	[36]
Herbicide	Atrazine, prometryn	[37]
Organic dye	Methylene blue, rhodamine B, congo red, acid Red 88, methyl orange	[38]
Pharmaceutical	Amoxycillin, ibuprofen, ciprofloxacin, omeprazole	[39]
Perfluoroalkyl substance	Perfluorooctanoic acid, perfluorooctane sulfonate	[40]
Microplastic	Polyvinylchloride, polyethylene, polypropylene	[41]

**Table 2:** Some MNP-based systems for remediation and bioremediation approaches.

Nanomaterial-Based System	Remediation Approach	Pollutant Treated	Ref.
AgNPs-PCBMA nanocomposite	Membrane filtration	Protein/Bio-fouling	[65]
Osmium NPs on polypropylene hollow fiber membranes	Membrane filtration/redox	p-nitrophenol and 10-undecylenic acid	[66]
Au/Bi_2_WO_6_ nanocomposite	Photocatalysis	Benzylic alcohols and Cr(VI)	[67]
Pd-Ag (NPs)/macroPSi heterostructure	Photocatalysis	Methylene blue	[68]
amid-p(Mac-co-AN)-M (M: Cu, Co) microgel	Catalysis	Nitrophenoles and cationic and anionic organic dyes	[69]
PHNA vesicle/AuNPs	Catalysis	4-nitrophenol	[70]
Cellulose-AgNPs composite	Photocatalysis	Bio-fouling and methylene blue	[71]
Cu-Ni hybrid NPs	Photocatalysis	Crystal violet dye	[72]
Karaya gum crosslink poly(acrylamide-co-acrylonitrile)@AgNP hydrogel	Adsorption	Crystal violet	[73]
Ag-Cellulose Acetate impregnated on polypropylene fibers membranes	Membrane filtration	H_2_S and C_2_H_5_SH	[74]
PU micelle/Ag NP clusters	In-situ extraction and detection (SERS)	Thiabendazole, phosmet and acetamiprid	[75]
rGO@AuNPs nanocomposite	In-situ detection and bioremediation	Cd^2+^, Pb^2+^, Cu^2+^ and Hg^2^	[76]
FeNiNPs@corncob-activated carbon	Photo-Fenton catalysis	Rhodamine B	[77]
PdNPs embedded over chitosan/γMnO_2_ microspheres	Catalysis	2-nitroaniline, 4-nitrophenol, 4-nitroaniline, 4-nitro-o-phenylenediamine, congo red, methylene blue, methyl orange, methyl red, and rhodamine B	[78]

**Table 3:** Some MONP-based systems for remediation and bioremediation approaches.

Nanomaterial-Based System	Remediation Approach	Pollutant Treated	Ref.
Fe-Ti-Mn composite oxide	Photocatalysis	As(V) and As(III)	[87]
ZnO@TiCN nanourchin	Thermochemical	Cr(VI)	[88]
PAMAM dendrimers with G4-OH cores immobilized on TiO_2_	Chelation	Cr (III), Cu(II) and Ni(II)	[89]
GO-MO nanocomposite	Inner-sphere complexation	Cd(II) and Cu(II)	[90]
Fe_3_O_4_@GOCMC core-shell structured composite bead	Adsorption	Phosphate and nitrate ions	[91]
PSB/Fe_3_O_4_/biochar composite	Removal and biodegradation	COD, phosphate and nitrate ions	[92]
TiO_2_-OP@Fe_3_O_4_ composite	Photocatalysis	Rhodamine B, Methylene blue, Congo Red and Cr(VI)	[93]
n–decanol membrane–10–undecylenic acid–iron oxide NPs	Liquid membrane	Silver and lead ions	[94]
Ethylene propylene diene monomer sulfonate impregnated membranes with propylene hollow fiber impregnated magnetic particles	Membrane filtration	Aluminum ions	[95]
Magnetite NPs, biochar and graphite alginate beads	Adsorption/bioremediation	Azo blue dye	[96]
Magnetic shell cross-linked knedel-like NPs	Adsorption	Crude oil	[97]
MNP-PEA-OmpA and MNP-PEA-OmpA-Laccase bionanocompounds	Bioremediation	Oil/water emulsions and crude oil	[98]
PET and sugarcane bagasse ash/Fe^3+^	Adsorption	Naproxen	[99]
Chitosan/Fe_2_O_3_/NiFe_2_O_4_	Adsorption	Methyl green	[100]
Chitosan/hydroxyethyl cellulose gel immobilized polyaniline/CuO/ZnO	Adsorptive-Photocatalytic	Congo red	[101]

**Table 4:** Carbon-nanomaterial based systems for remediation and bioremediation approaches.

Nanomaterial-Based System	Remediation Approach	Pollutant Treated	Ref.
CNTs, nanofibers and vermiculite based nanosponge	Absorption	Oil	[116]
PANi/CNT composite	Microbial fuel cell	COD	[117]
MWCNT/Ppy composite	Microbial fuel cell	Phenol and COD	[118]
AQS/Fe_3_O_4_/CNTs and HA/Fe_3_O_4_/CNTs composite	Biocatalysis	Cr(VI) and methyl orange	[119]
Graphene aerogel/Fe_3_O_4_/polystyrene composite	Absorption	Crude oil	[120]
L-GO/ZnO NPs composite	Microbial fuel cell	Pb^2+^	[121]
Graphenized sand-based composite	Absorption	Hg^2+^	[122]
Ni-BDC@GO nanocomposite	Adsorption	Methylene blue	[123]
TZB-Gr composite nanofiber	Photocatalysis	Methylene blue and rhodamine B	[124]
P25-GR composite	Photocatalysis	Phenanthrene, fluoranthene, and benzo[a]pyrene	[125]
TiO_2_ composite based on zinc porphyrin-covalently functionalized fullerene [C60]	Photocatalysis	Phenol and methylene blue	[126]
ZrO_2_ NPs on GO supported peptide/cellulose binary nanofibrous membrane	Membrane filtration	Fluoride ions	[127]
Cyclodextrin modified GO@FeNP composite	Adsorption	Oxytetracycline	[128]
CNTs/carbon xerogel hybrid loaded Fe–Ni	Adsorption	RY160 dye	[129]
Activated carbon based on shea residue (*Vitellaria paradoxa*)	Adsorption	Hydroquinone and resorcinol	[130]

**Table 5:** Silica-nanomaterial based systems for remediation and bioremediation approaches.

Nanomaterial-Based System	Remediation Approach	Pollutant Treated	Ref.
Ppy/HMSNs hybrid	Adsorption	Cr(VI)	[153]
Pd@h-mSiO_2_ nanotubes	Catalysis	4-nitrophenol and 4-chlorophenol	[154]
Mesoporous silica NPs and magnetic GO	Adsorption	Sulfamethoxazole antibiotic	[155]
Fe_3_O_4_-MWCNTs@SiO_2_ nanocomposite and laccase	Biocatalysis	Eriochrome Black T, Acid Red 88, and Reactive Black 5	[156]
SBA-15 mesoporous silica and laccase	Biocatalysis	Protocatechuic acid, ferulic acid, sinapic acid and caffeic acid	[157]
Fe_3_O_4_@SiO_2_@Ru hybrid magnetic composite	Photocatalysis	Methyl orange and methyl red	[158]
Cu-Z-GO-M composite	Adsorption	Methylene blue	[159]
nZVI/Ni@FZA composite	Adsorption	Cr(VI) and Cu(II)	[160]
T/MZ composite	Photocatalysis	Metoprolol	[161]
Zeolite, GO and laccase bio-nanocompound	Biocatalysis	Direct Red 23	[162]
Zeolite coated by *Pseudomonas veronii* cells on xanthan gum-based biopolymer	Biocatalysis	Hg^2+^	[163]
Cellulose fibers/zeolite-A nanocomposite	Adsorption	Organic and inorganic Se ions	[164]
ODTMS modified halloysite	Bioremediation	Crude oil	[165]
Sodium alginate/halloysite/hemp hurd	Adsorption	Methylene blue	[166]
PU foam coated with POS@HNT	Absorption	Chloroform and dichloroethane	[167]
Iron−clay−cyclodextrin composite	Catalysis	Bisphenol A, carbamazepine and perfluorooctanoic acid	[168]
Chitosan-clay and laccase nanobiocomposite bead	Biocatalysis	Anthrax quinone dye, azo dye of and diazodye	[169]
Biocomposite membranes of chitosan with montmorillonite and kaolin	Adsorption	Cu(II)	[170]
